# Turkish imams and their role in decision-making in palliative care: A Directed Content and Narrative analysis

**DOI:** 10.1177/02692163221095200

**Published:** 2022-06-06

**Authors:** George Muishout, Nuray Topcu, Anne de la Croix, Gerard Wiegers, Hanneke WM van Laarhoven

**Affiliations:** 1 Department of History, European Studies and Religious Studies, Amsterdam School for Historical Studies, University of Amsterdam, Amsterdam, The Netherlands; 2 Independent Researcher; 3 Research in Education, Amsterdam UMC, Faculty of Medicine, Vrije Universiteit Amsterdam, Amsterdam, The Netherlands; 4 Department of Medical Oncology, Cancer Center Amsterdam, Amsterdam University Medical Center, University of Amsterdam, Amsterdam, The Netherlands

**Keywords:** Muslims, imams, palliative care, decision-making, withholding treatment, terminating life-sustaining treatment

## Abstract

**Background:**

Muslims are the largest religious minority in Europe. When confronted with life-threatening illness, they turn to their local imams for religious guidance.

**Aim:**

To gain knowledge about how imams shape their roles in decision-making in palliative care.

**Design:**

Direct Content Analysis through a typology of imam roles. To explore motives, this was complemented by Narrative Analysis.

**Setting/Participants:**

Ten Turkish imams working in the Netherlands, with experience in guiding congregants in palliative care.

**Results:**

The roles of Jurist, Exegete, Missionary, Advisor and Ritual Guide were identified. Three narratives emerged: *Hope can work miracles, Responsibility needs to be shared*, and *Mask your grief*. Participants urged patients not to consent to withholding or terminating treatment but to search for a cure, since this might be rewarded with miraculous healing. When giving consent seemed unavoidable, the fear of being held responsible by God for wrongful death was often managed by requesting fatwa from committees of religious experts. Relatives were urged to hide their grief from dying patients so they would not lose hope in God.

**Conclusion:**

Imams urge patients’ relatives to show faith in God by seeking maximum treatment. This attitude is motivated by the fear that all Muslims involved will be held accountable by God for questioning His omnipotence to heal. Therefore, doctors may be urged to offer treatment that contradicts medical standards for good palliative care. To bridge this gap, tailor-made palliative care should be developed in collaboration with imams. Future research might include imams of other Muslim organizations.

**What is already known about the topic?**

- To Muslims it is important that medical decisions are in accordance with Islamic values.
- In life-threatening illness, Muslims ask imams for religious advice on medical decision-making. So far, it is unknown how imams view and perform this task in palliative care.

**What this paper contributes?**

- Imams advise the faithful not to consent to withholding or terminating treatment based on diagnostics because they feel this does not align with belief in God’s omnipotence and will.
- Imams are reluctant to advise patients to consent to termination of treatment for fear that all Muslims involved will be held accountable for someone’s death by God in the afterlife.
- Fatwas by Muslim expert committees play an important role in shaping medical decision-making in palliative care.

**Implications for practice, theory or policy**

- Palliative care tailored to the cultural religious needs of Muslim minorities must be developed.
- Implementing adequate palliative care for Muslim minorities requires sustainable collaboration with imams and their congregations.

## Introduction

With a share of five per cent of the total population Muslims are one of the largest religious minorities in Europe.^1^ Many of them have a Turkish background.^2^ A characteristic of terminal illness in Muslims is an intensified focus on religion to cope with this profound event.^3–6^ In this context, religion not only provides beliefs concerning death but also functions as a normative framework in medical decision-making.^7–11^ Particularly in palliative settings, Muslims, like Christians and Jews, call on their religious leaders to learn the religious permissibility of treatment proposals.^4,12–14^ They contact mosque-based imams who fulfill similar roles as pastors and rabbis.^7,12,15^ In addition to leading the five daily prayers and preaching the Friday sermon, imams serve their congregations by conducting rites of passage (involving birth, marriage, and death), providing spiritual care, religious education and answering questions about Islamic law, including those about medical decision-making.^12,16–18^ As yet, no research has been done into the way imams in the West perform their task in palliative care and their role. Hence, there is a knowledge gap regarding their contribution to the dynamics of doctor-patient relationships. To better understand medical decision-making in palliative care among Muslims, it is therefore important to investigate how they are guided by their imams.

## Methods

### Design

We undertook an interview study and qualitatively analyzed the gathered interview data to shed light on the attitudes and motivations of imams in the performance of palliative care. We aimed to discover *which roles* they see for themselves and *how they construct* these roles in the stories they tell. The two sub-aims warranted two methodological approaches. For the first aim we used a Directed Content Analysis. This is characterized by thematic structuring of texts using a pre-existing theoretical framework.^19^ The second aim concerning construction of their role in their stories was explored through Narrative Analysis. Narrative Analysis conceives personal accounts as narratives in which individuals give meaning to experienced reality to construct their identity in interaction with their audience.^20^

### Setting

The study took place in the Netherlands where Muslims comprise 5% of the population.^15^ The majority are of Turkish and Moroccan descent; they make up 37% viz 36% of the Muslim population and have their own religious organizations.^17,21,22,23^

### Study population

Since our research team included a native speaker of Turkish, we focused on Turkish imams.

### Recruitment

Imams of Turkish mosques in the four largest Dutch cities and surrounding municipalities were purposefully selected according to the following criteria: (1) being an experienced and full-time professional imam; (2) having received formal Islamic training in their country of origin; (3) having regularly advised and spiritually guided congregants in palliative settings.^24^

### Ethical considerations

Participants gave written consent after being informed about the purpose of the study. Due to the sensitivity of the issues, all agreed to encoded use of data. Names were therefore deleted in the transcripts. The Academic Medical Center’s Medical Research Ethics Committee ruled that the Dutch Medical Research Involving Human Subjects Act (WMO) did not apply (Ref: W21_192 # 21.209app.).

### Data collection

Semi-structured interviews were conducted in Turkish between February 2018 and June 2019 by NT, of which four together with GM. All interviews took place in the mosques in which the participants served as imams, in space suitable for undisturbed conduct of in-depth interviews. Participants were asked to talk about a case that involved withholding or terminating treatment they had been professionally involved in. Following an interview guide, they were encouraged to explore their thoughts and feelings (Table 1). Interviews lasted one to two hours and were recorded and transcribed verbatim. Participants were sent their transcript by email and given the opportunity to voice second thoughts. Once they agreed with the content, interviews were translated into Dutch by NT. Translations were checked for accuracy by an independent native Turkish speaker with an academic proficiency in Dutch. Based on this, final versions were determined.

**Table 1.** Interview guide.

**Main questions** 1. Would you like to take a case in mind concerning withholding or terminating treatment in which you were involved as Imam? 2. What was going on in this case (background)? 3. Who were involved in it? 4. What part did you play in it? 5. Have you given any advice to those involved? If so, what was your advice? 6. How did you come to that advice? 7. How did those involved react to this? 8. Have you been in contact with those involved afterwards? 9. How do you look back on this case? If something like that happened again, would you act / advise the same? ***The role of doctors in withholding or terminating treatment*** 10. How do you view the role of the doctor in withholding/withdrawing treatment? 11. What does a treatment proposal or decision from a doctor mean to you? What if a doctor proposes to terminate treatment or to withhold it? 12. Does a doctor’s treatment proposal or decision affect the way you advise those involved? 13. Do you yourself consult with doctors about what they recommend to their patients regarding withholding or terminating treatment? Or do you do this in other ways, for example through an interpreter or intermediary? ***Withholding or terminating treatment*** 14. What is in your opinion withholding and withdrawing treatment? 15. How do you view withholding and withdrawing treatment? 16. Can you explain how you underpin your view (from an Islamic perspective)? 17. Which religious authorities are important to you in medical decision-making? 18. Are there certain statements of religious scholars that guide you concerning withholding or terminating treatment? 19. How do you view refusing treatment? 20. What do you think about the role of “quality of life” in medical decision-making? Are there things you find important to say but have not been addressed?

### Data analysis

The research team provided written comment on the translated transcripts once data collection started. After six interviews no new variations were found. To generate a richer dataset, four more interviews were conducted. The results confirmed our previous findings. GM analyzed the content using Ajouaou’s typology as an analytical framework.^16^ Using religious terms commonly known among Muslims, this typifies the basic functions imams perform in accordance with the expectance of congregation members who appeal to them for pastoral care. The typology distinguishes jurist (applier of Islamic law), exegete (interpreter of the Quran and the traditions of the Prophet Muhammad), missionary (preaching correct religious doctrine and the corresponding way of life), adviser (giving practical advice for dealing with difficult situations), prayer leader (leader of the five daily obligatory prayers), guide in rituals and blessings (pastor at prayers and rituals at rites of passage) and teacher (teaching religious skills such as performing obligatory prayer and reciting the Quran) (Table 2). The performance of these roles is situational and represents complementary and often overlapping modes that imams use to approach a pastoral issue. With a view to interpretative reliability, NT randomly coded five interviews through this framework, after which it was concluded that these results matched those of GM. Subsequently GM structured the stories within the interviews applying Labov’s approach, which assumes narratives have distinctive features. These are the *Abstract* (what the story is about), *Orientation* (what, when and to whom something happened), *Complicating Action* (what happened after this), *Evaluation* (the moral of the story), *Resolution* (the outcome) and optional *Coda* which takes the audience back to the present moment (and. . .here we are). The *Evaluation* is pivotal. It concerns language that storytellers use to make value judgments about their characters’ actions (“in doing so, she did the right thing”) or fragments in which they step out of their story to comment on it for the purpose of conveying its moral to their audience (“You should know that it is beyond my responsibility to speak out on such things.”). Carefully evaluating *how* stories are told provides insight into the deeper meaning, motives and values the narrator wants to convey to his audience.^25^ After finalizing the results, AdlC and GM created a typology of the narratives.

**Table 2.** Summary of the different roles of imams.

Role	Description
Jurist	Applier of Islamic religious law, that is, gives answers to the question: is something morally allowed or not?
Exegete	Interpreter of the Islamic theological sources in contingent issues. (In the context of palliative care this particularly concerns themes such as illness, treatment and (a good) death.)
Missionary	Preacher of correct religious doctrine and the corresponding way of life (commanding right and forbidding wrong). This is done by distilling values from the Quran, the tradition of the Prophet Muhammad (sunna) and Islamic tradition in general; the believer is called upon to relate these to sickness and death, inwardly and outwardly.
Advisor	Advisor in tackling the question: what must be done practically to deal with a specific problem?
Prayer Leader	Leader of five daily joint obligatory prayers
Guide of Rituals and Blessings	Spiritual guide in rituals and blessings in the religious Islamic tradition, in the transition from earthly life to the hereafter.
Teacher	Religious teacher (includes teaching skills for ritual practice such as reciting the Quran and performing obligatory prayers).

Source: Adapted from Ajouaou.^16^

## Results

Twenty-seven candidates were approached. Twelve were not selected due to lack of experience with palliative care. Five declined to participate due to personal considerations or policies in their organization prohibiting their participation. Ultimately, 10 participated in the study (Table 3). We came across the roles of Jurist, Exegete, Missionary, Adviser and Guide in Rituals and Blessings.

**Table 3.** Participant characteristics.

Characteristics	Number
Country of birth	Turkey *n* = 9 Netherlands *n* =1
Mean age	43.9 (range 28-55)
Organisation	Transnational Turkish *n* = 9 Local Turkish *n* = 1
Highest level of education	Master *n* = 2Bachelor *n* = 6 post-secondary vocational education *n* = 1High school *n* = 1
Mean years of residing in the Netherlands	14.8 (range 3-27)
Mean number of years of work experience	15.6 (range 7-26)

### Jurist

Congregants appealed to participants with the question whether a proposal to terminate treatment was in accordance with Islamic law. In exercising this jurist role, participants urged patients to continue treatment. Muslims are not allowed to interfere in life and death because God decides on this.

“You should continue until the end. Do not discontinue based on arguments such as: it is painful and harms the patient. These are all useless things. Just keep going. . .Even though the patient screams in pain day and night. . . I cannot interfere in divine matters.” *(Participant 8)*

Continuing treatment meant that when a doctor declared all treatment options to have been exhausted, a second opinion had to be requested. To ensure proper understanding of afterlife religious consequences of decision-making, a Muslim doctor was strongly preferred for this task. What was striking was that withdrawing treatment was associated with killing the patient.

“Yes, well. . .. of course, you do. . . don’t misunderstand me. The judgments of both Muslim and non-Muslim doctors are valuable because they are both medical experts. A judgment or proposal from a non-Muslim doctor must of course also be respected. But when it comes to ending a human life, we must think a hundred times. This involves ethical and religious responsibility. In Islam, killing a person is like killing all people on earth. It is a terrible act with unimaginably great responsibility . . . and sin. . . I wouldn’t like to bear that as an imam. I don’t know if a non-Muslim doctor is aware of this. . . aware of Islamic ethics and values and their consequences in the afterlife. But a Muslim doctor does. That makes all the difference.” *(Participant 10)*

In cases where patients could not be kept alive with life support most participants transferred the role of jurist to *fatwa councils* within their organizations by asking these religious expert committees whether it was permissible to *unplug*. The imam then communicated the committee’s affirmative response to the relatives. This allowed the imam and family members involved to avoid interfering in divine matters and thereby becoming responsible for the religious consequences of decision-making.

“You should absolutely consult the Committee so that neither you nor I bear any responsibility for it. I discuss those things with you and, although I will give you a likely answer, I refer you to the Committee. It is the Committee that takes the decision.” *(Participant 1)*

### Exegete

Four participants exercised their role of exegete by establishing causal relationships between their interpretations of the Quran, which they consider to be God’s word, and what they believed to be the divinely determined outcome of the disease process. They transferred their exegesis to the patient’s family by advising them on *how to act* in medical decision-making. Therefore, they interpreted a positive course of the disease as God’s reward for their endurance. This meant that a patient’s recovery after following their advice to disagree with the proposal to discontinue treatment was ascribed to their having acted according to God’s command.

“It *was on a Friday . . . My wife called me. I heard in her voice that she was almost crying, as I am now (participant speaks in a trembling voice, GM). I asked what had happened. She said. . .* “Ms. . . called me. Her mother has got better.” *I asked her how. My wife replied*: “She can’t *walk but she got better . . . she can talk. . .I thought. . . I started to* philosophize *and said to myself: if they had unplugged her based on my advice or that of another imam or someone else. . . that would have been a great sin. I thanked God for this. When we act, with the Quran as reference . . . then we are sustained by verses like*: “He leads them to a right path (Quran 5:16, GM).” *I said to the* patient’s *daughter*: “Your mother will heal even more because you have acted according to God’s command. You have sought healing and have expressed your fear of responsibility. Believe me . . . God is going to help you.” *(Participant 2)*

### Missionary

 In discussing life-threatening illness, participants positioned themselves toward the interviewers as missionaries, preaching how Muslims should deal with this in terms of correct beliefs and attitudes. Central to their view on the right Muslim attitude was *never to give up hope.* Unlike with unbelievers, there was no question of hopelessness among Muslims when doctors did not expect their patients to recover. Therefore, the participants saw not initiating treatment on grounds of futility as un-Islamic. Good Muslims must believe in God’s omnipotence to cure.

“The bottom line is that we shouldn’t forget that God has absolute power, that He is omnipotent. When we philosophize about why someone loses hope, we must question his faith in God. Doing nothing to initiate treatment or not starting treatment at all by saying it is useless is un-Islamic.”*(Participant 6)*

### Adviser

Participants provided practical advice on *how to cope* with life-threatening illness. This advice was complementary to their religion-based advice, viz aimed at prolonging life and avoiding responsibility for the patient’s death by not agreeing to terminate treatment. Family members were advised to take heart from similar cases in which patients recovered against all odds, since doctors could be wrong.

“We know and have seen that there are those who have returned to normal life - perhaps not as comfortably as before - who had been said to never, ever show a sign of life other than a last breath. This is proof enough for us to not pull the plug. Moreover, we see that doctors are often mistaken. And we should not forget that we would eventually be killing someone without valid reason or by mistake. I said we should look at it from this perspective.” *(Participant 2)*

### Guide in Rituals and Blessings

Participants performed rituals aimed at guiding the dying in their transition to the afterlife. Unlike the other roles that concerned religious responsibilities, this role was directed at serving the needs of the dying. Reciting a specific chapter of the Quran and gently encouraging the dying to utter the Islamic creed were ways of easing the burden on the patient. Moving the dying to utter the Muslim testimony of faith served as a reaffirmation of faith before the soul left the body.

“It concerned a patient, an old aunt, at the end of her life. We went there. As just said . . . considering the verse “You alone we worship . . .” (Quran 1:4, GM). She was at her last stage of life. You just know that. For these situations, some criteria have been set out in Islamic jurisprudence. . . Yes, what are these things? What does our Prophet advise us to do in these situations? The thing you do should is mentally ease the burden on the patient. Read chapter Yā Sīn (chapter 36 of the Quran, GM). Whether the patient hears that or not. . . only God knows. Try to remind her of the tawḥīd (God’s oneness, GM), shahāda (the Muslim testimony of faith, GM) and ṣalawāt (sending blessing upon the Prophet Muhammad, GM) without burdening her. Especially tawḥīd and shahāda, because perhaps the moment of departure of the soul from the body is the final test. I have tried to perform all these rituals to the maximum with this patient.” *(Participant 1)*

### Narrative analysis

To enrich the Directed Content Analysis, we studied the stories told by the participants. The aim was to reveal the underlying meaning using Labov’s approach. Therefore, we focused on the *evaluation*, that is the story elements that show how the narrator wants it to be interpreted by the audience. Three narrative types were distinguished: *Hope can work miracles* (participant 2, 3, 6, and 7), *Responsibility needs to be shared* (participant 1, 5, 8, 9, and 10) and *Mask your grief* (participant 4) (Table 4).

**Table 4.** Overview narratives and summary of categories.

Participant	Story type	Abstract	Orientation: what, when and with whom did something happen?	Complicating Action: then what happened?	Evaluation: the moral or message of the story	Resolution: The outcome or result	Optional Coda: Takes the audience back to the present moment
1	Responsibility needs to be shared	A brain dead woman is in the hospital. The imam visits her in his capacity as transition counselor, which service he provides by reciting the Quran.	*When*: Four months before the interview. *Who*: dying woman, relatives, imam, doctors. *What*: woman who is diagnosed with irreversible brain damage, palliative decision-making *Where*: the hospital	At the suggestion of the doctors, relatives intend to have the life support equipment unplugged. They ask for the Imam’s religious opinion. Due to the great religious responsibility, the Imam absolutely does not want to take a position here. Therefore, he calls the fatwa committee of his organization and passes the question on to them.	Firstly, Muslims are to respect the sanctity of life. Therefore, the responsibility to answer questions about discontinuing treatment in the context of terminal illness is hardly bearable from a religious point of view on an individual level. Instead, this burden should be borne by a collective of members of a *fatwa* committee. Secondly, since God is omnipotent, Muslims must by definition, even in the event of a fatal diagnosis, *believe in* and therefore *hope for* a cure.	The *fatwa* committee agrees *to pull the plug*, which brings the family mental relief.	N/A
2	Hope can work miracles	An old woman with severe intestinal ailment using life support equipment is medically given up by the attending doctor, who therefore proposes to discontinue treatment and send her home. One of her three daughters invites the Imam, through his wife, in his role as judge to answer the question: Does our religion allow this unplugging?	*When: not explicitly mentioned Who: patient, daughter of the patient, wife of the imam, imam, attending doctor. What: old woman with severe intestinal ailment, palliative decision making Where: the* daughter’s *house, the hospital, the house of the Imam*	The imam advises the daughter not to go along with the doctor’s proposal but to look for a Muslim doctor for a second opinion instead.	Central to the story is the concept of *hope*. Discontinuing treatment means losing faith in God and thus, in a deeper sense, loss of faith. If someone makes the *right choice* by continuing to seek healing, God provides help by making it come true. Thanks to this *choice* others are protected from the grave religious sin of being responsible for someone’s death.	The daughter agrees with the imam’s advice and puts it in into practice. After a second opinion with a Muslim doctor who starts to treat the woman, she shows hopeful signs of recovery.	The imam wonders how the old woman is doing at the moment and intends to make an inquiry after the interview.
3	Hope can work miracles	A severely ill mosque- goer, unconscious, dependent on life- support equipment, is visited in the hospital by his group of friends from the mosque and the imam.	*When*: not explicitly mentioned *Who*: severe sick mosque- goer, group of friends, son of the patient, Imam *what*: severe sick, unconscious mosque-goer, palliative decision-making *Where*: the hospital, the mosque	Through the grapevine the imam receives the patient’s son’s question how to respond to the doctor’s proposal to *pull the plug*. The imam instinctively advises not to agree with the doctor’s proposal.	God and not man determines the moment when someone dies. In this context, the Imam’s advice not to *pull the plug* on the sick patient, who then does not die but resumes his life, should be seen as divine inspiration.	The son adopts the advice of the imam after which the patient miraculously recovers and returns to his old habit of frequently visiting the mosque.	Until now the former patient keeps visiting the mosque.
4	Mask your grief	A man goes on pilgrimage with the Imam to Mecca. During the trip he has some physical complaints. After returning he undergoes examination in the hospital and is diagnosed with colon and pancreatic cancer associated with a life expectancy of six weeks.	*When: not explicitly mentioned Who: dying patient, family members, imam What: dying patient, spiritual care Where: Mecca, the hospital*	The imam, informed by his family about his medical condition in advance, visits the patient in his role as *Guide in rituals and blessings* knowing that he is dying.	When death is inevitable, only an inner and outer attitude of hope and complete reliance on God, which are reflections of one’s faith, can bring salvation.	The patient and all family members are stimulated to adopt a hopeful attitude with complete trust in God. In this context, family members are urged to mask their grief in the presence of their dying loved one.	N/A
5	Responsibility needs to be shared	A premature baby with insufficiently developed lungs is dependent on ventilation equipment. The attending doctor considers the child not viable in the medium term. Therefore, he considers continuing treatment as prolonging the child’s suffering and proposes pulling the plug.	*When: not explicitly mentioned Who: baby, father, mother, Imam, attending doctor What: premature baby with underdeveloped lungs, palliative decision-making Where: the mosque, the hospital.*	The father of the baby visits the imam with the question whether his religion allows them to *pull the plug*. Before giving a fatwa, the Imam visits the hospital to be personally informed by the doctor about the baby’s medical situation. The doctor explains that life- sustaining treatment will only prolong the baby’s suffering.	Asking an imam for *fatwa* about permissibility of *pulling the plug* is essentially intended to throw off the responsibility - religiously and mentally- from the questioner in his/her final decision. In this particular case, the imam carries the responsibility of his *fatwa* with him forever, which is presented as very heavy personal burden.	The imam tells the parents that there are no religious barriers to agreeing with the doctor’s proposal. The parents decide to go along with the doctor’s decision and convey this to the doctor. In the presence of the parents and the imam, the plug is pulled, after which the child dies.	The imam bears the responsibility of having agreed to pull the plug.
6	Hope can work miracles	The imam is approached by telephone by a member of his congregation whose father is in a coma and who asks him to pray for his sick father.	*When*: not explicitly mentioned *Who*: male patient, sun, wife of the patient, imam, doctor *What*: comatose patient, palliative decision making *Where*: unclear (the location of the imam and the son of the patient is unclear at the moment of the call), the hospital	After two weeks the imam is called again by the son. He tells him that the doctors want to *pull the plug*, by arguing that recovery with a reasonable quality of life is deemed impossible. The imam discusses the patient’s medical condition face to face with the doctor, after which he advises the son not to agree with the doctor’s proposal.	Fearing God and hoping in God are qualities that distinguish Muslims from unbelievers. Therefore, hope, arising from faith in God’s omnipotence, may bring recovery from illness deemed incurable by non- religious doctors based on their worldly standards. This is a phenomenon that is even confirmed by these doctors themselves.	The son takes the advice of the imam, after which the patient awakens after forty days from his coma and undergoes a miraculous recovery.	N/A
7	Hope can work miracles	A woman is asked to agree to the doctor’s suggestion to have the plug pulled on her husband because continuation would be futile. She cannot agree to this and refuses.	*When*: not explicitly mentioned *Who*: severely sick mosque-goer, wife, children, imam, doctor *What*: patient unable to breathe independently, palliative decision-making *Where*: the mosque, the hospital	The woman visits the imam for the purpose of seeking confirmation for the correctness of her refusal, her choice.. The imam then confirms her choice.	Doctors should do everything to keep someone alive. Therefore, they have no right to intervene in the process of dying by *pulling the plug.* The miraculous healing of patients prove the doctors to be wrong (and the imam to be right).	The patient recovers miraculously and resumes his old habit of praying in the Mosque.	Until now the former patient visits the mosque to perform his prayers.
8	Responsibility needs to be shared	A mosque-goer calls the imam with the request to visit his seriously ill father-in-law in the hospital to recite the Quran for him. This is what happens, to the satisfaction of the patient and the family.	*When*: 2018 *Who*: patient, wife, children, grandchildren, son-in-law, imam *What*: dying patient, palliative decision-making *Where*: hospital, the home of the family	The patient’s situation gradually worsens, after which the doctors propose to *pull the plug* so that the patient can die at home. The imam is asked by the family for advice on how to respond to the proposal. He advises not to agree with it. The advice is acted upon by the family after which the patient lives another three weeks.	Firstly, giving religious advice about discontinuing treatment requires great caution because it involves the risk of being personally responsible for the death of someone else. Secondly, prolonging terminal life for a short period of time through medical support may contribute to gradually accepting the impending death of a loved one.	After a month, the family invites the imam to their home to recite the Quran as part of the ritual remembrance of the deceased. The family expresses its gratitude for the imam’s advice because it has allowed them to be with their loved one a little bit longer.	N/A
9	Responsibility needs to be shared	The imam is called by a son or brother of a terminal patient who asks how to deal with the doctor’s suggestion to discontinue life support.	*When*: not mentioned *Who*: terminal patient, son or brother, imam *What*: terminal patient, palliative decision-making *Where*: unclear (it concerns a telephone call)	The imam advises not to agree to the proposal. Instead, the family should show patience and keep hoping in God.	God determines life and death. Therefore, Muslims should use all medical means because stopping or withholding treatment is equivalent to losing hope in God. For Imams this implies that they cannot advise other than to continue treatment for as long as possible.	The imam then verifies his answer with the fatwa committee, which he always does in such cases, which confirms the rightness of his advice.	It is unclear whether the patient is still alive or not. However, the imam says that after this kind of advice, which he always gives in similar situations, patients often turn out to be alive months later.
10	Responsibility needs to be shared	The imam is called by a male family member of a terminally ill patient who has been hospitalized for a long time asking whether their religion allowed them to agree with the doctors’ proposal to discontinue treatment. The imam does not answer immediately, but promises to do so after peer consultation	*When*: not explicitly mentioned/ some time ago *Who*: terminal patient, family member, imam, fellow imams *What*: terminal patient, palliative decision-making *Where*: unclear (it concerns several phone calls)	In consultation with fellow imams, it is collectively decided to advise that everything should be done to continue treatment since human life must be protected as much as possible. The imam contacts the family member and informs him of their *fatwa*.	God owns and decides life and death. Contributing to the death of a person is a very grave, (almost) unpardonable sin. Therefore, neither imams nor laypeople should take the responsibility for choosing to discontinue treatment resulting in death.	The family member responds positively to the advice of the Imam.	It is unclear what ultimately happened to the religious advice. In retrospect, the imam thinks that the man had already intended to focus on continuation of treatment. Therefore, his role consisted only of confirming this position.

### Typology of narratives

#### Hope can work miracles

Doctors propose withholding or terminating treatment because they consider recovery to be impossible. After following the imam’s advice to disagree with this, the patient shows miraculous signs of recovery. The message is that God is omnipotent, so nothing is impossible. If Muslims continue to hope by searching for a cure, basing that hope in a firm belief in God, He may reward them by making it happen.

“At the initiative of a Moroccan doctor, they transferred her to another hospital. After two days they heard from the Moroccan doctor that there was hope, the hope of being able to open her bowels. To be honest, I got more curious and said to my wife: ‘Give them a call and ask them about the latest situation.’ Madam (the patient’s daughter, GM) said that her joy had made her forget to inform us. . . ‘My mother’s intestines have opened. This Moroccan doctor made an extra effort. . . a Muslim doctor has done a lot.’ My previous advice to the daughter was . . . I must mention this . . . that according to verse 53 of sūrah Al-Zumar (Quran chapter 39, GM) we must not despair of God and according to sūrah Yusuf (Quran 12: 87, GM) those who despair of God are the disbelievers. And so, we should not forget that it is God who grants healing.” *(Participant 2)*

#### Responsibility needs to be shared

Agreeing to terminate treatment is associated with religious burden. This burden stems from the relationship between terminating treatment and the subsequent death of the patient. The possibility of such an association—and thus being responsible for the chain of events—implies a potential contribution to the patient’s death. Consequently, those involved fear being held liable by God in the afterlife for the capital sin of culpable death. Therefore, advice on terminating treatment cannot be taken by imams alone but must be shared.

“Yeah. . . withholding treatment. . . terminating treatment. Right at that point, a sane imam or a believer with knowledge of Islam in his own individual capacity cannot say yes and agree with such a doctor’s decision. I’ve been through many situations like this. You have arrived at the last crucial, deciding moment. From where you should get the last word, the all-determining word. The sentence: yes. . . treatment can be terminated; the plug can be pulled. Islamic community Millî Görüş (Turkish religio-political Islamic organization, GM) has a fatwa commission. A decision of this committee, therefore, is not of an individual person but a committee consisting of at least twelve expert (religious, GM) scholars. Diyanet (the Turkish presidium for Turkish affairs, GM) also has a fatwa committee. I can refer such situations to these committees. A verdict must come from these kinds of committees. So, as a mosque imam I cannot take such a responsibility. Because, may God protect us, even if the chance is one in a thousand of a wrong pronunciation, an imam cannot take that responsibility. For in the Quran, there is a verse that says: whosoever slays a soul – unless it be for another soul or working corruption upon the earth – it is as though he slew mankind altogether. . . (Quran 5: 32, GM). This could confront us with being guilty of killing humanity in its entirety. As an imam, I absolutely cannot assume such a great responsibility.” *(Participant 1)*

#### Mask your grief

When death is inevitable, only God can bring salvation by giving eternal live in heaven. Qualifying for such salvation requires an attitude in which the dying fully trusts God. Therefore, religious guidance should focus on stimulating the dying to reinforce this trust by affirming his/her belief in God.

“Guiding him spiritually and reminding him of religious things was also something I wanted to do as an imam. The only thing that would keep him going was faith. Surrender to God, trust in God, the only one who could help. Belief in God. . .There is nothing else that keeps man on his feet.” *(Participant 4)*

To prevent the dying from becoming ineligible for salvation by giving up hope in God, family should radiate hope and hide their grief in direct contact with the dying.

“I explained to them that a family has many tasks. Being a family during the disease process requires a particular course. The family should be aware of this. Their job is to keep the patient from giving up hope in God. Although the family is affected by the patient’s illness, they should not show this to the patient but radiate hope. They should not let the patient notice a negative influence (of his sickness, GM) on their body language (and should hide, GM) their psychological breakdown, as much as possible.” *(Participant 4)*

## Discussion

### Main findings

This study shows how imams construct their roles in palliative care. Our Directed Content Analysis informs the Narrative Analysis by showing *how* transformation of this task is modulated through its role content. In turn, the Narrative Analysis informs the Directed Content Analysis by revealing motives behind religious advice and demonstrating its function within the story types. Our Directed Content Analysis revealed two patterns in response to proposals to withhold or terminate treatment. Where the first aims to maximize treatment, the second revolves around dealing with religious responsibility. The pattern aimed at maximizing treatment is narrated through the narrative *Hope can work miracles*. Modulated through the role content of *exegete*, the *how* of the story explains healing as God’s reward for striving for maximum treatment. This is complemented through the role of *adviser* by pointing out misdiagnoses in which patients recovered. The *missionary*-linked content, the *why* of the story, underscores God’s omnipotence. Consequently, diagnostics that imply absence of cure should be rejected. Therefore, medical decision-making is constructed as a matter of faith in which not giving treatment (for reasons of hopelessness or futility) represents *disbelief* while hope for miraculous healing by striving for maximum treatment objectifies *true belief in God*.

Miracle-driven belief in cancer patients is known to reduce the ability to understand prognostics.^26^ Patients’ relatives sometimes believe that God’s omnipotence manifests itself through intervention in the course of the disease.^27,28^ In our data such belief—attached to striving for maximum treatment, aimed at *inviting God* to perform a miracle, as it were—is not just optional but presented as a condition for being a faithful Muslim. In a taxonomy that classifies God-attributed miracles, unshaken evocations concern a category in which belief in miracles is embedded in religions with worldviews enjoying supreme authority.^29^ Since Muslims are committed to consistency between religious norms and treatment proposals affirmed through their imams, this is likely to affect clinical practice.^30,31^ Projected onto our results, it evokes an image in which religion-based belief and evidence-based medicine are antithetical.^26,32–34^

The second pattern, which concerns religious responsibility, is narrated through the narrative *Responsibility needs to be shared*. The *how* of the story concerns the probability of being held accountable by God in the hereafter that comes with the role of *jurist.* This is due to interference with the moment of death by agreeing to withhold or terminate treatment. The *missionary-*role-linked message is that God disposes over life and death. Therefore, those who agree to terminate treatment run the risk of unauthorized access to His domain.

The tendency among participants to pass on questions about medical decision-making to fatwa committees is a present-day form of the traditional Islamic way of dealing with applied ethics. In it, imams are part of hierarchical structures in which fatwas are no longer the domain of the individual mufti.^16,35,36^ They are now part of the collective effort of expert committees.^35,37–39^ Our data show that participants made exclusive use of such fatwas issued by their own organizations. Imams disseminate viewpoints on decision-making in palliative care that have been determined top down among their congregants.^2,22,40–42^ Some of our participants issued fatwas themselves. This fits within a development in which imams fulfill tasks because the institutions that provide these in the country of origin are lacking.^16,18,43^

### Strengths and limitations

Participants were aware that the interviewers were Muslims. We believe that this shared religious identity made it easier for them to speak about the sensitive nature of palliative care. However, our sample predominantly consisted of imams representing transnational organizations. Therefore, one might wonder to what extent our data are personal views or representations of the formal Islamic views and policies of these organizations. We believe this characteristic constitutes both strength and limitation. A strength is that our research provides insight into how imams as representatives of organizations contribute to the shaping of palliative care. A limitation is that is not always clear where personal views were expressed. Conversely, we think that they saw representing normative Islamic views as obvious, as this was linked to their professional identity. Another limitation is that our sample is limited to Turkish imams. Therefore, different results might be observed in follow-up studies including imams from other Muslim minorities.

### What this study adds

Our study shares similarities with earlier research among lay-Muslims about end-of-life decision-making. A common denominator is the view that God is omnipotent and disposes over life and death.^44–47^ For some this means that treatment may be withheld or terminated to avoid suffering because God ultimately decides the moment of death, while this is very hard for others since it undermines belief in God’s omnipotence to cure.^44–47^ Our findings suggest that imams emphasize God’s omnipotence to cure by pushing for maximum treatment. Striking in this context was the impact of fear among participants. Taking medical decisions in palliative care for family members is known to evoke negative emotions, including the fear of interfering with God’s plan.^48–51^ It is this fear that motivated participants to refrain from issuing statements about withholding or terminating treatment. This suggests that they view issuing fatwas in such cases as synonymous with becoming decision-makers.

Our findings reveal a gap between palliative care tailored to individual patient needs and an afterlife-orientated approach by imams that stresses the religious responsibilities of all involved.^44,45,52^ This is worrisome since research points to unequal access and less favorable outcomes of palliative care among minorities.^52,53^ What is needed is tailor-made palliative care embedded within cultural-religious frameworks that meets the specific needs of Muslim patients and their social system.^52^ To develop this requires policy and concrete action from healthcare providers to build sustainable relationships with imams and their congregations aimed at working together in the interest of the Muslim patient.^53^

## Conclusions

The imams’ insistence on maximizing treatment in their advice to patients’ relatives in medical decision-making in palliative care is intertwined with the fear of being held accountable by God for intervening with His omnipotence and will. As a result, clinical practice may face negative attitudes among Muslims toward proposals to withhold or terminate treatment which are at odds with medical views on good palliative care. To bridge this gap, palliative care policies tailored to the needs of Muslims need to be developed in close cooperation with imams and their congregations. To broaden the understanding of imams’ contributions to palliative care, future research should include imams of different ethnic backgrounds.
